# Growing Teratoma Syndrome and Peritoneal Gliomatosis

**DOI:** 10.1155/2011/123527

**Published:** 2011-04-07

**Authors:** H. Mrabti, I. El Ghissassi, Y. Sbitti, M. Amrani, H. Hachi, H. Errihani

**Affiliations:** ^1^Department of Medical Oncology, National Institute of Oncology, Rabat, Morocco; ^2^Department of Histopathology, National Institute of Oncology, Rabat, Morocco; ^3^Department of Surgery, National Institute of Oncology, Rabat, Morocco

## Abstract

The growing teratoma syndrome (GTS) is defined as a detection of an enlarged mass during or after chemotherapy treatment for germ cell tumor. We report a case of an 18-year-old girl treated for growing teratoma syndrome after chemotherapy for malignant germ cell tumor of the ovary associated with peritoneal gliomatosis. Chemotherapy induced normalisation of alpha-fetoprotein rate whereas there was an enlargement of the mass. Subsequent complete resection was performed, and the patient remained in good control for 60 months. This clinical picture suggested the diagnosis of “GTS”. This syndrome can lead to confusion with progression or relapse of a germ cell tumour because of increase in tumour volume during chemotherapy, so it is important to recognize it.

## 1. Introduction

The growing teratoma syndrome (GTS) is defined as a detection of a benign enlarged mass during or after chemotherapy treatment for germ cell tumor. The diagnosis is based on three criteria [1]: increasing size of tumor volume and metastasis during chemotherapy for malignant germ cell tumor, normalization of markers who were initially high during or after chemotherapy, and the presence in the histological examination of the postchemotherapy surgical specimen of a mature teratoma without evidence of malignancy. 

This entity was first described by Logothetis et al. in 1982 [2]. Other authors had described it under the name of “chemotherapeutic retroconversion”.

This syndrome occurs in 1.9 to 7.6% of testicular nonseminomatous germ cell tumors [3], but it appears exceptionally in patients treated for ovarian germ cell tumors (OGCTs). The peritoneal gliomatosis is defined by the presence of peritoneal implants, corresponding to nodules of mature glial tissue in patients with ovarian teratoma. 

We report a case of an 18-year-old girl treated for growing teratoma syndrome after chemotherapy for malignant germ cell tumor of the ovary associated with peritoneal gliomatosis.

## 2. Observation

An18-year-old girl, with no previous health problems, no previous pregnancy, an age of first menstruation of 13 years, presented with a 5-month history of abdominal pain and increased abdominal volume. Physical examination revealed a huge abdominopelvic mass, and ultrasound and CT scan revealed an abdominopelvic mass of 22 × 18 cm in diameter, starting from the right ovary, without lymphadenopathy or liver metastasis. Preoperative tumor markers were high with an alpha-fetoprotein (AFP) rate of 210 ng/mL (normal value <6 ng/mL), beta-human chorionic gonadotrophin at a normal rate (2.45 mu/mL), Ca 125 rate of 30 u/mL (normal value <35 u/mL), and lacticodehydrogenase was at 326 u/L (normal value < 480 u/L). 

Laparotomy revealed a huge right ovarian mass with multiple peritoneal granules measuring between 0.2 and 0.5 cm. A total hysterectomy, oophorectomy, and omentectomy were performed. Histological examination of the ovarian mass (weight = 1880 g) revealed a teratomatous tumor, with grade 2 immature areas, showing neuroepithelial elements. The contra lateral ovary was not involved.

Peritoneal granulations consisted of a mature glial tissue. Examination of peritoneal fluid did not reveal suspicious lesions. Postoperatively, the rate of AFP was 76.98 ng/mL. 

The patient had an immature teratoma on histopathology. The histopathological specimen analyzed did not show aspects of yolk-sac and/or embryonal carcinoma. The coexistence of an elevated AFP and immature teratoma concluded to a diagnosis of a nonseminomatous ovarian germ cell tumor. 

**Figure 1:**
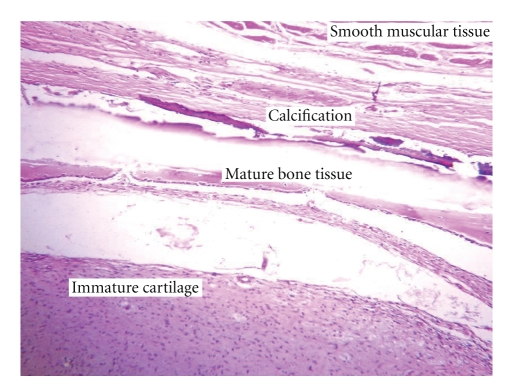
Postchemotherapy histology: mature teratoma (hematoxylin and eosin stain ×10).

**Figure 2:**
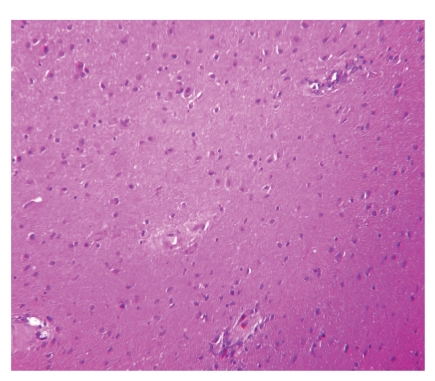
Postchemotherapy histology: mature glial tissue (hematoxylin and eosin stain ×20).

Chemotherapy combining cisplatin 100 mg/m^2^ on day 1 and etoposide 120 mg/m^2^ on days 1-2-3, every 21 days, was administered for 6 cycles with normalization of AFP from the 4th cycle (1,34 ng/mL); other markers were still unremarkable. Moreover postchemotherapy pelvic CT (6 months after surgery) revealed a voluminous pelvic mass 25 × 21 × 11.5 cm containing calcifications and fatty areas. A second laparotomy was performed, revealing a huge abdominopelvic mass and multiple peritoneal implants, and the surgical procedure had consisted of an optimal cytoreduction. Surgical pathology consisted of benign mature teratoma with no neuroectodermal component (Figure 1), associated with peritoneal gliomatosis (Figure 2). The patient remained in good control 60 months after the second intervention with regular monitoring of markers and abdominopelvic computed tomography scan.

## 3. Discussion

GTS and “chemotherapy retroconversion”, defined as conversion of a metastatic immature teratoma into a metastatic mature teratoma as a result of chemotherapy are two names denoting the same entity; the concordance of these two entities has been recently confirmed by Amsalem et al. [4]. 

The GTS rarely occurs in association with OGCT but was more commonly described in males treated for testicular nonseminomatous germ cell tumors. 

Two etiopathogenic mechanisms for the occurrence of this syndrome were discussed [1]: selective destruction of the malignant component of immature teratoma as a result of chemotherapy and persistence or progression of chemoresistant benign mature teratomatous elements. The second hypothesis is spontaneous differentiation of malignant cells into benign tissues. 

Tangjitgamol et al. [5] had found, after a review of the English literature, 30 cases of GTS. Only one feature seemed to predispose to the occurrence of a GTS: the presence of mature teratoma at the initial histological examination, found in 62.5% of patients who developed ovarian GTS.

This syndrome may also cause confusion with a progression or recurrence of a germ cell tumor because of the increase in tumor volume during chemotherapy, hence the importance of recognizing it. Moreover patient's eventual treatment and prognosis are highly dependent on the timing of diagnosis because detection of GTS in a delayed stage results in a more extensive surgical dissection with a higher associated risk of adjacent organ injury.

In the case studied, the patient had initially a huge abdominal mass with a high rate of AFP, confirming the malignancy of the germ cell tumor. The diagnosis of GTS was made in view of the increase in tumor size after chemotherapy and normalization of AFP. This diagnosis was confirmed by the postchemotherapy pathological examination revealing a benign mature teratoma, without malignant germ cells. Teratoma did not include a neuroectodermal component.

An optimal cytoreduction is the recommended treatment of GTS, because of the risk of obstructive complications and rapid increase in tumor volume, which could lead to inoperability [3, 5]. Chemotherapy and radiotherapy do not seem to have a role in the treatment of GTS because of its benignity [5]. 

Careful monitoring is required due to the high risk of recurrence until 10 years after initial diagnosis [6–8], particularly in cases with significant residual tumor. Recurrence will also be eligible for surgery. 

The prognosis of this benign entity remains favorable, with a survival of up to nine years in completely resected cases [9]. 

About 95% of ovarian germ cell tumors are represented by pure teratoma, in comparison to 4% of testicular germ cell tumors. Since ovarian teratomas are derived from benign germ cells, immature elements represent the evolution of a malignant clone [10]. Immaturity usually manifests as immature neuroepithelium that develops within a pre-existing teratoma [10].

Prognosis for immature teratoma of the ovary is related to stage and grade of the tumor. The 2-year disease-free survival for grade 2 immature teratoma is about 50%. Recurrence can be minimized by postoperative adjuvant therapy with bleomycin, etoposide, and cisplatin (BEP) if the tumor is of grade 2 or 3. Second aggressive debulking is limited to patients having a growing teratoma syndrome after immature teratoma [11].

Immature ovarian teratomas are associated with gliomatosis peritonei, a favorable prognostic finding if composed of completely mature tissues.

The peritoneal gliomatosis (PG) is a very rare entity, defined by the implantation of glial tissue in the peritoneal cavity, omentum, and abdominal lymph nodes. PG occurs almost exclusively in combination with an ovarian teratoma, whatever its grade [12]. 

Two theories have been proposed regarding the pathogenesis of PG. One of them postulates that the PG is derived from teratoma associated with the relocation of cells from the primary tumor through the capsular defect (spontaneous or surgical) or by lymphogenous metastatic spread [13]. The other theory suggested [14–19] that glial implants develop from normal cells having undergone a metaplastic process in response to an unknown endogenous or exogenous neoplastic stimulus. The latter seems to be the most appropriate.

 Molecular studies have been performed to better understand the relationship between an ovarian teratoma and PG. Best et al. [15] performed a molecular analysis of a patient diagnosed with immature ovarian teratoma and GP and demonstrated mutually exclusive genetic differences among the tumors, establishing the neoplasms as genetically distinct from each other, representing multiple independent tumors rather than true tumor recurrence or spread. Ferguson et al. [16] studied DNA samples extracted from ovarian teratoma and glial implants from two patients. They exploited the unique genetic characteristic of many ovarian teratomas (containing a duplicated set of maternal chromosomes and are thus homozygous at polymorphic microsatellite loci), contrasting with DNA from matched normal or metaplastic tissue (containing genetic material of both maternal and paternal origin and are heterozygous at many of these same microsatellite loci). In these two cases, all implants and normal tissue showed heterozygosity at each of three microsatellite loci on different chromosomes, whereas the teratoma showed homozygosity at the same microsatellite loci, indicating that glial implants in GP often arise from cells within the peritoneum, presumably pluripotent Müllerian stem cells, and not from the associated ovarian teratoma.

By performing the same molecular analysis, Kwan et al. [17] concluded that PG is genetically unrelated to the associated teratoma but is probably derived from nonteratomatous cells, such as through metaplasia of submesothelial cells.

The association of GTS and PG was previously described by Shefren et al. [20], who reported the case of a patient with grade III immature teratoma associated with extensive peritoneal implants of mature glial tissue. The implants of glial tissue were present during both the initial and the second-look laparotomy, performed after chemotherapy. The “chemotherapeutic retroconversion” seems to participate in the development and progression of the PG, because GTS and PG are both benign glial implants either in the peritoneum or ovary. 

A review of the literature, reported by Chou et al. [21], had found 65 cases of PG, which have favorable prognosis after surgical treatment. 

Treatment of PG is the complete surgical resection, which has two objectives: confirmation of diagnosis and therefore the exclusion of malignancy, but also the prevention of malignant transformation of residual lesions. 

Complete excision is often impossible, given the extent of the lesions, hence the importance of close monitoring of residual lesions, using imaging such as CT scans. 

The presence of a PG at the initial laparotomy may be predictive of the occurrence of a GTS after chemotherapy of a germ cell tumor of the ovary [22], hence the value of an optimal initial surgery to prevent disease progression. A long-term monitoring is also required.
